# Survival of Composite Resin Restorations of severely Decayed Primary Anterior Teeth retained by Glass Fiber Posts or Reversed-orientated Metal Posts

**DOI:** 10.5005/jp-journals-10005-1344

**Published:** 2016-06-15

**Authors:** Ali Vafaei, Bahram Ranjkesh, Henrik Lovschall, Leila Erfanparast, Mohammad A Jafarabadi, Sina Ghertasi Oskouei, Flemming Isidor

**Affiliations:** 1 Assistant Professor, Department of Pediatric Dentistry, School of Dentistry, Tabriz University of Medical Sciences, East-Azarbayjan, Tabriz, Iran; 2 Fellow, Section of Dental Pathology, Operative Dentistry and Endodontics, Department of Dentistry, Aarhus University-Health, Midtjylland, Aarhus, Denmark; 3 Associate Professor, Section of Dental Pathology, Operative Dentistry and Endodontics, Department of Dentistry, Aarhus University-Health, Midtjylland, Aarhus, Denmark; 4 Assistant Professor, Department of Pediatric Dentistry, School of Dentistry, Tabriz University of Medical Sciences, East-Azarbayjan, Tabriz, Iran; 5 Assistant Professor, Department of Statistics and Epidemiology, Tabriz University of Medical Sciences, East-Azarbayjan, Tabriz, Iran; 6 Dentist, School of Dentistry, Tabriz University of Medical Sciences East-Azarbayjan, Tabriz, Iran; 7 Professor, Section of Prosthetic Dentistry, Department of Dentistry Aarhus University-Health, Midtjylland, Aarhus, Denmark

**Keywords:** Deciduous teeth, Dental restoration failure, Glass fiber post, Reversed-orientated prefabricated metal post, Survival.

## Abstract

**Aim:** The aim of this study was to compare the survival of composite resin restorations retained by glass fiber posts or reversed-orientated (upside-down) metal posts in severely decayed primary anterior teeth after 6, 12, and 18 months.

**Materials and methods:** A total of forty-four 3- to 5-year-old children with bilateral severely decayed primary maxillary canines were included. Patients were treated under general anesthesia. After pulpectomy, an intracanal post was seated in the primary maxillary canine on each side: either a glass fiber post or a metallic post in reversed orientation and teeth restored with light-cured composite. Survival rate of each technique was evaluated at predetermined follow-ups and data were analyzed with McNemar’s test (α = 0.05).

**Results:** The difference in survival of restorations retained by two types of posts was not statistically significant in clinical and radiographical evaluations after 6, 12, and 18 months. The survival rate of reversed-orientated metal and glass fiber posts after 18 months was 81.1 and 67.6% respectively (p = 0.14).

**Conclusion:** Reversed-orientated metal post did not show lower clinical survival compared with glass fiber posts in 18-month follow-up. Hence, reversed-orientated metal post can be considered as a potential method to obtain retention for composite restorations in severely decayed primary anterior teeth.

**How to cite this article:** Vafaei A, Ranjkesh B, L0vschall H, Erfanparast L, Jafarabadi MA, Oskouei SG, Isidor F. Survival of Composite Resin Restorations of severely Decayed Primary Anterior Teeth retained by Glass Fiber Posts or Reversed-orientated Metal Posts. Int J Clin Pediatr Dent 2016;9(2):109-113.

## INTRODUCTION

Restoration of primary anterior teeth with extensive caries in children with low cooperation is determined as a challenging treatment for a dentist.^1^ Children’s esthetic, masticatory function efficacy, phonetics, space maintenance, and prevention of malocclusion until physiologic exfoliation of primary teeth increase the restoring tendency of severely decayed primary anterior teeth.^2,3^ However, insufficient coronal tooth structure of severely decayed primary teeth endangers the retention and endurance of the restorations. Therefore, the use of different postplacement techniques to obtain retention in pulpectomized teeth can increase the survival of the restoration. On the contrary, physiologic root resorption of primary teeth is the main limiting factor for postplacement in the primary dentition,^1^ which precludes the use of the entire root canal length. Therefore, the coronal third of the canal is commonly used for retention acquisition in such circumstances.^1,4,5^

Metal posts, biologic posts, omega-shaped stainless steel orthodontic wires, polyethylene fiber posts, and glass fiber posts are routinely suggested retention techniques for primary teeth restoration.^6^ Conventional use of prefabricated metallic posts is a fast, inexpensive, and simple technique, but unesthetic appearance and interference with physiologic resorption limit the usage in primary dentition.^7,8^ Use of omega-shaped orthodontic wires has been introduced as a fast and simple technique with good adaptability to canal walls of primary tooth, but early detachment of restoration and fractures of thin root canal walls are predictable.^9-12^ In recent years, more attention has been focused on the use of fiber-reinforced composite posts, where prefabrication, mechanical, and chemical bonding to the final restorative material, reduction in the risk of root fracture, and absence of discoloration are some of the possible advantages for fiber-reinforced composite posts.^13^ Nevertheless, high cost for a deciduous tooth, technique sensitivity, and time-consuming treatment procedures are the main concerns, particularly in case of an uncooperative child.^4^

Recently, use of reversed-orientated (upside-down) metal posts has been advocated for intracanal retention in the restoration of severely decayed primary anterior teeth. A post space no more than 3 mm of the root length and quadrangle core placement of the metal post in the prepared space may provide adequate retention for longer lasting restorations.^4,14^ The aim of the present study was to compare the survival of composite restorations retained by glass fiber posts or reversed-orientated metal posts in severely decayed primary anterior teeth 6, 12, and 18 months after treatment.

## MATERIALS AND METHODS

A total of 44 children among the children who were referred to the Department of Pediatric Dentistry, Dental Hospital at Tabriz University of Medical Sciences for dental treatment under general anesthesia because of lack of cooperation were included in the study. The inclusion criteria were: (1) 3- to 5-year-old children with early childhood caries, (2) no medical consideration, (3) presence of bilateral severely decayed primary maxillary canines with minimum one-fourth remaining coronal teeth structure, (4) hopeless primary maxillary incisors indicated for extraction, (5) present or restorable molar teeth, (6) normal overjet, (7) no malocclusion, and (8) sound root of canine teeth with no more than one-third apical resorption in comparison with adjacent teeth. Informed consent was obtained from each participant’s parent before treatment. The study was complemented as a randomized controlled clinical trial with a split-mouth design. The study protocol was approved by the ethics committee of Tabriz University of Medical Sciences.

Children were treated under general anesthesia in a single treatment session to receive complete dental rehabilitation. After clinical and radiographic evaluations of the maxillary canine on both sides, root treatment was commenced. All caries were removed and a full pulpectomy under isolation with cotton rolls was done. The root canal was prepared using a sequence of three consecutive endodontic files #30-45 (Maillefere, Kerr, Orange, CA, USA) and constant irrigation with physiologic saline. Canals were dried with paper points and obturated with zinc oxide eugenol paste (ZOE; Dentsply, Caulk, Milford, DE, USA).

In reversed-orientated metal post group, the post space was prepared using fissure bur (Dentsply, diamond bur 109/008, USA) to match the quadrangle head of the metal post. A short, prefabricated, gold-plated metal (brass) screw post (ProduitsDentaires, SA, Switzerland), which fitted to the coronal segment of the root, was selected. The fit of the post head in the quadrangle preparation and correct placement of the post were checked before cementation. Adequate incisal space for the composite resin restoration was secured by adjusting the length of the post.^4^ The metal post was cemented into the canal in reversed orientation (upside-down) with glass ionomer cement (Fuji I, GC International, Tokyo, Japan).

In glass fiber post group, 4 mm of the canal was coronally depleted of any trace of ZOE on the canal walls by 1.1 mm diameter post space preparation bur (Itena, Paris, France). The most apical part of the prepared space (1 mm) was filled with glass ionomer cement (Fuji I, GC International, Tokyo, Japan) to avoid any composite setting impairment by ZOE cement. The length of the glass fiber post (Itena, Paris, France) in the canal was determined and the adjusted post was placed in the canal for length confirmation. Before cementation, the post was cleaned with ethanol and thoroughly air-dried. Adequate space for composite resin restoration was checked. The walls of the post space were etched with 37% phosphoric acid (3MESPE, Saint Paul, MN, USA), rinsed, and dried. Afterward, the primer and adhesive (Optibond, Kerr, Orange, CA, USA) were applied for the entire post space and cured for 20 seconds. The post space was filled with flowable light cure composite^13^ (Vertise Flow, Kerr, Orange, CA, USA) and cured for 60 seconds^13^ with Bluephase® light-emitting diode curing light unit (Ivoclar Viva-dent, Schaan, Liechtenstein).

To restore the crown of the teeth in both groups, the remaining tooth substance was etched and rinsed. The primer and adhesive (Optibond, Kerr, Orange, CA, USA) were applied to the etched tooth structure and the threaded part of the metal post and light-cured for 40 seconds. A thin layer of universal opaque flowable composite resin (Universal Opaque, Revolution Formula 2, Kerr, Orange, CA, USA) was placed over the metal post to prevent the metal post shade shining through the restoration and cured for 20 seconds. The coronal restoration was incrementally placed using nanohybrid dental composite (Herculite Ultra, Kerr, Orange, CA, USA) with A1 shade.^4^ After filling, the occlusal interferences in all the lateral and anterioposterior extrusions of the mandible were checked and restorations were finished and polished. We accomplished all required treatments of whole dentition, including extraction of incisors and restoration of molars, in the same session. Parents were instructed in oral hygiene of the child and a low cariogenic diet was recommended.

All patients were evaluated after 6, 12, and 18 months. Survival or failure of the restored tooth complex was evaluated clinically and radiographically. Each restoration was judged as failure if it met one of the following criteria: (1) loss of tooth, (2) restoration loss because of restoration and post absolute debonding, (3) restoration loss because of canal and post debonding, (4) post fracture, (5) periapical radiolucency, and (6) pathologic root resorption. One pedodontist accomplished all restorative procedures during the study and another pedodontist, who was blinded to the treatment, carried out the follow-ups.

## STATISTICAL ANALYSIS

The absolute and relative effect size of differences was reported by both relative difference and relative risk with 95% confidence interval according to CONSORT statement. The data were analyzed using McNemar’s test by statistical software STATA version 11.0 (StataCorp LP, College Station, TX, USA), with significance level of 0.05.

## RESULTS

During the course of the study, there were a total of seven drop-outs (Flow Chart 1) of 5, 0, and 2 subjects in 6-, 12-, and 18-month follow-ups respectively, leaving a total of 37 lasting cases for final analysis (Table 1).

In a 6-month follow-up (n=39), the survival rate for reversed-metal post and glass fiber post was 97.4 and 89.7% respectively. Reversed-post group showed one failure as complete post and restoration debonding, while the post still remained in the canal, whereas in the glass fiber post group, four failures were seen: Two cervically fractured posts and two complete debonding of the restoration and glass fiber post from the root canal. No periapical lesion or root resorption was observed in the two groups.

**Flow Chart 1:**
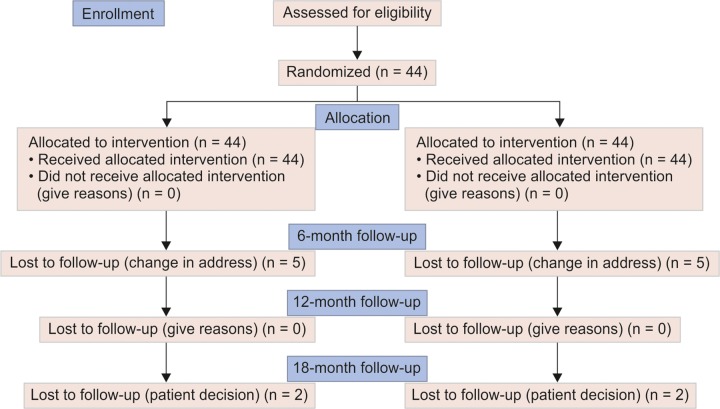
Flow diagram of the study

**Table 1:** **Table 1:** Cumulative failure numbers and survival rate for two treatment methods evaluated after 6, 12, and 18 months

				*Reversed-* *metal* *post*		*Glass* *fiber* *post*		*Risk* *difference* *(95% CL)*		*p-value*	
6 months		Failures		1/ 39		4 / 39		7.7% (- 5.8		0.179	
		Survival		97.4%		89.7%		to 21.2)%			
12 months		Failures		4 / 39		10 / 39		15.4% (- 3.9		0.069	
		Survival		89.7%		74.4%		to 34.6)%			
18 months		Failures		7 / 37		12 / 37		13.5% (- 6.2		0.144	
		Survival		81.1%		67.6%		to 33.2)%			

Considering cumulative measures in a 12-month follow-up (n = 39), three more failures were found in reversed-metal post group (a total of four failures), causing 89.7% survival rate in comparison with 74.4% in fiber glass post treatment group. All observed failures in reversed-metal post group were complete restoration and post debonding, while the post was present in the canal. None of the teeth restored with this technique displayed periapical lesions or root resorption in radiographic evaluation. In glass fiber post group, we observed six more failures: Four fractured posts in the cervical area and two complete debonding of the restoration and glass fiber post from the root canal (a total of ten failures accumulatively). One of the latter cases exhibited internal resorption along with a periapical radiolucency in the radiographic evaluation. The mean risk difference between two groups after 6 and 12 months was 7.7 and 15.4% respectively. However, the risk difference between two treatment groups was not statistically significant (p > 0.05).

A total of 18 months after treatment (n = 37), we found three more failures in reversed-metal post group; all failures were with complete restoration and post debonding. In glass fiber post group, two more failures were seen: One fractured fiber from the coronal segment and one total fiber post debonding from the root canal. The latter demonstrated a periapical radiolucency.

The cumulative survival rate after 18 months of treatment for reversed-post and fiber glass post was 81.1 and 67.6% respectively. The reversed metal posts showed 13.51% (-6.2, 33.2) higher survival rate than glass fiber posts; however, analysis of data did not reveal any significant differences (p > l0.05).

## DISCUSSION

Use of intracanal retention for the restoration of deciduous teeth is mainly indicated when remarkable coronal structure of teeth has been lost. Hence, mechanical retention acquisition from the root canal after pulpectomy procedures may provide successful restorations with satisfactory resistance against masticatory forces.^12^

Prefabricated metallic posts provide a fast, easy-to-perform, inexpensive, and less technique-sensitive conventional use in comparison with glass fiber posts. However, both unesthetic appearance, resulting from the color of the post and the physiologic resorption of the primary teeth root, limit their application.^7,8^ In the reversed metal post technique, the quadrangular core of the metallic post is cemented into the most 3 mm coronal part of the canal that will not interfere with the physiologic resorption of the primary tooth root.^4^ Therefore, a major obstacle of the metallic posts usages in pediatric dentistry is diminished.

Glass fiber posts, carbon fiber posts, Kevlar fiber posts, and polyethylene fiber posts are some available forms of fiber-reinforced composite posts with acceptable esthetic.^12^ In this study, the survival rate of restoration reconstructed with fiber post and reversed metal post after 18 months was 67.6 and 81.1% respectively; however, the risk difference (13.5%) was not statistically significant between the two groups. Eshghi et al reported 90% of reversed metal posts and 84% of fiber posts were acceptable for maxillary primary incisor restoration after 12 months according to the evaluation criteria of the study. However, the rate of restoration retention (presence of restoration) was reported 100 and 90% for reversed metal post and fiber posttreatment group respectively.^14^ Our finding in this study for the survival of restoration using two techniques showed slightly more failure rate after 12 months: 89.7% for reversed-metal post and 74.4% for fiber post without statistical significant difference. Failure criteria, study design, and treatment circumstances can describe the discrepancies of the two studies. We evaluated the survival rate of two techniques in restoration of the canine teeth, where the most parafunctional and eccentric movement loads are prone to be applied, whereas Eshghi et al treated maxillary primary incisors. On the contrary, they compared the two techniques in a randomized clinical trial study, whereas our study was accomplished in a split-mouth design. Therefore, the effects of confounding factors, such as different masticatory forces and other parafunctional habits may be more unified with the split-mouth design. Moreover, extraction of incisors and restoration of molar teeth in our study may minimize the occlusal load pattern diversity in each patient. Nevertheless, the results in the two studies did not show statistically significant difference using two techniques after 12 months.

In this study, glass fiber posts fractured cervically in seven cases, where the higher shearing load is predicted to be exerted. On the contrary, none of the reversed metal posts illustrated the same failure mode during the study period. Lower flexural strength and lower stiffness of glass fiber post in comparison with metal posts could elucidate this failure type for glass fiber post.

Interestingly, five glass fiber posts deboned from the root canal, whereas this type of failure was not observed among teeth which restored with reversed metal post. Even in the case of restoration loss in reversed metal post group, all posts remained still inside the canal. It has been shown that reversed metal post can gain more mechanical retention *vs* glass fiber post.^15^ Thus, using reversed metal post, more mechanical retention may be obtained rather than fiber post, which post retention is majorly provided by cement in 3 mm length. Considering the most failure mode in the two evaluated methods, remaking potency of the restoration seems to be more feasible among the teeth treated with a reversed metal post than a glass fiber post, as all observed failures in reversed metal post were complete post and restoration debonding. However, use of reversed metal post may cause more stress concentration surrounding the root area, we did not observe any root fracture during 18 months. Presence of root fracture was not also reported in a previous study.^14^

Regarding the esthetic of restoration, reversed placement of the metal post core into the root canal can provide sufficient space for the bulk of restorative composite resin. Furthermore, a thin layer of opaque flow composite over the metal screw can reduce the visibility of the post through the restoration and enhance the final esthetic appearance. Eshghi et al^14^ showed acceptable color match and translucency for primary maxillary incisal teeth restored with reversed metal post after 1 year.

## CONCLUSION

The use of prefabricated reversed-orientated metal post and glass fiber post can have a substantial role in the rehabilitation of severely decayed primary anterior teeth. Based on the results of the present study, reversed-orientated metal post did not exhibit lower clinical survival *vs* glass fiber posts during 18 months. Therefore, reversed-orientated metal post with acceptable clinical survival may be considered as a potential method to obtain retention for composite restorations in severely decayed primary anterior teeth. Further studies with more sample size and longer follow-up period are needed.
